# Protein-bound polyphenols create “ghost” band artifacts during chemiluminescence-based antigen detection

**DOI:** 10.12688/f1000research.10622.2

**Published:** 2017-05-26

**Authors:** Nathalie Plundrich, Mary Ann Lila, Edward Foegeding, Scott Laster

**Affiliations:** 1 Plants for Human Health Institute , North Carolina Research Campus, North Carolina State University, North Carolina, USA; 2 Department of Food, Bioprocessing and Nutrition Sciences, North Carolina State University, North Carolina, USA; 3 Department of Biological Sciences, North Carolina State University, North Carolina, USA

**Keywords:** western blot artifacts, egg white proteins, enhanced chemiluminescence, ghost band, green tea polyphenols, horseradish peroxidase, protein-polyphenol interactions

## Abstract

Antigen detection during Western blotting commonly utilizes a horseradish peroxidase-coupled secondary antibody and enhanced chemiluminescent substrate. We utilized this technique to examine the impact of green tea-derived polyphenols on the binding of egg white protein-specific IgE antibodies from allergic human plasma to their cognate antigens. Our experiments unexpectedly showed that green tea-derived polyphenols, when stably complexed with egg white proteins, caused “ghost” band formation in the presence of horseradish peroxide. This study suggests that caution should be taken when evaluating polyphenol-bound proteins by enhanced chemiluminescence Western blotting using horseradish peroxidase and demonstrates that protein-bound polyphenols can be a source of “ghost” band artifacts on Western blots.

## Introduction

Western blotting has been used extensively to identify and quantify relative amounts of specific proteins in complex mixtures. Proteins are identified using antigen-specific primary antibodies followed by various enzyme-coupled secondary antibodies. Commonly used conjugated enzymes are alkaline phosphatase and horseradish peroxidase (HRP) ^1^. HRP is more popular due to its stability and smaller size, which allows for conjugation of multiple HRP moieties per secondary antibody and increased sensitivity ^2^. Avidin-biotin systems can also be used to amplify reactivity and luminol-based enzyme substrates are commonly used to create a visible chemiluminescent signal.

We recently evaluated peanut protein-polyphenol aggregate particles for their binding capacity to peanut-specific plasma IgE from allergic patients using complementary assays, including chemiluminescence-based Western blotting ^3^. Previous studies have shown “ghost” bands on some blots. In the present study, we demonstrate that protein-bound polyphenols can cause “ghost” band artifacts during chemiluminescence-based antigen detection. We investigated the binding of IgE antibodies to hen egg white proteins complexed with green tea-derived polyphenols. For detection on the blots, we used primary antibodies from allergic human plasma, secondary biotin-coupled goat anti-human IgE, avidin-HRP, and an enhanced luminol substrate. Results showed that HRP is required for “ghost” band formation. Caution should be taken when evaluating polyphenol-bound proteins by enhanced chemiluminescence Western blotting.

## Methods

### Materials

Precast mini TGX 4–20% polyacrylamide gels were purchased from BioRad (Hercules, CA, USA). Nitroblue tetrazolium and glycine were purchased from Sigma-Aldrich (Sigma-Aldrich, St. Louis, MO, USA). All other SDS-PAGE and immunoblotting reagents used are listed elsewhere ^3^. Egg white protein (EWP) was purchased from Sigma-Aldrich (St. Louis, MO, USA). Commercially available organic dry green tea leaves ( *Camellia sinensis* [L.] Kuntze) were provided by QTrade Teas & Herbs (Cerritos, CA, USA). Ground leaves were extracted and stored until further use as previously described ^1^. Extraction was performed for 2 h at 80°C.

### Preparation of egg white protein-green tea polyphenol aggregate particles

The total phenolic content in the green tea extract was determined (36.8 mg mL ^-1^ ± 0.26 mg mL ^-1^, see Table S1) according to the 96-well microplate-adapted Folin-Ciocalteu method by Zhang *et al.* ^4^ with modifications described by Herald *et al.* ^5^. The amount of extract (mL) and protein powder (g) required to generate dry, stable protein-polyphenol aggregate particles containing 5, 10, 15, 30, or 40% polyphenols after complexation was added together and mixed under constant agitation for 15 min at room temperature. Mixtures were subsequently frozen at -20°C and freeze-dried (FreeZone12, Labconco, Kansas City, MO, USA) to form stable protein-polyphenol aggregate particles.

### Nitroblue tetrazolium (NBT) staining to reveal polyphenols

Following transfer of proteins by electroblotting from unmodified EWP and aggregate particles to a polyvinylidene difluoride (PVDF) membrane, the membrane was briefly hydrated in 100% methanol. Subsequently, polyphenol-modified proteins were detected with NBT and glycinate as described by Hagerman [ 6; www.users.muohio.edu/hagermae/]. At alkaline pH, the catechol moiety of polyphenols catalyzes redox-cycling in the presence of glycinate, generating superoxide that reduces NBT to insoluble, visible formazan ^7^.

### SDS-PAGE and immunoblotting

Amounts of protein-polyphenol aggregate particles or unmodified EWP were normalized to provide 2 mg protein for SDS-PAGE. Samples were prepared in sample loading buffer containing 5% β-mercaptoethanol, resulting in 10 µg protein in 10 µL. Samples (10 µg protein/10 µL) were incubated for 5 min at 95°C, loaded onto a gel, run (40 min at 200 V), and then stained with Coomassie Brilliant Blue (CBB). The immunoblotting method used, including reagent sources, is described elsewhere ^3^. The following minor modifications were made: Pooled human plasma (containing polyclonal antibodies, among them egg white-specific IgE) from 7 egg white-allergic individuals (PlasmaLab International, Everett, WA, USA; 1:80; v/v) was used to bind antigens on the membrane. EWP-specific IgE levels ranged from 15.4 to 100 kU L ^−1^ as determined via ImmunoCAP (Phadia, Uppsula, Sweden). Biotinylated polyclonal goat IgG anti-human IgE (Kirkegaard & Perry Laboratory, Inc., reference no. 01-10-04, Gaithersburg, MD, USA; 1:8,000; v/v) and NeutrAvidin HRP conjugate (Thermo Scientific, Rockford, IL, USA; 1:20,000; v/v) were used to bind plasma antibodies.

In separate experiments, proteins in aggregate particles containing 15% polyphenols were blotted onto a PVDF membrane. The membrane was subsequently cut into strips and subjected to various combinations of immunoblotting reagents. Transferred proteins from unmodified EWP served as controls. The proteins from unmodified EWP were subjected to the full immunoblotting procedure.

## Results and discussion

### Protein distribution, NBT staining, and IgE binding capacity

**Figure 1.**
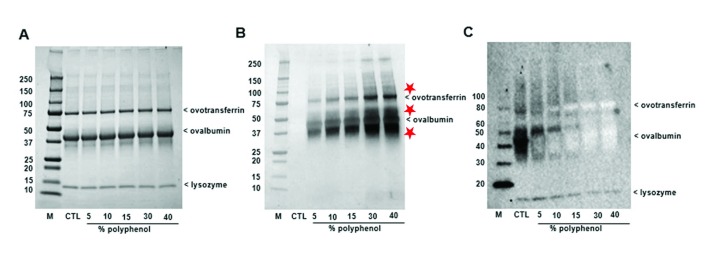
Protein distribution visualized by Coomassie Brilliant Blue staining (CBB), nitroblue tetrazolium (NBT) staining, and IgE binding capacity. ( **A**) SDS-PAGE of unmodified egg white protein (CTL) or egg white protein-polyphenol aggregate particles containing 5, 10, 15, 30, and 40% polyphenols and stained with CBB; ( **B**) Staining of green tea polyphenol-bound egg white proteins by NBT, following SDS-PAGE and subsequent electrophoretic transfer to a PVDF membrane; ( **C**) corresponding Western blot. Pooled human plasma from 7 egg white-allergic individuals was used to bind antigens on the membrane. Egg white-specific IgE levels ranged from 15.4 to 100 kU L ^−1^ as determined via ImmunoCAP (Phadia, Uppsula, Sweden). Biotinylated goat IgG anti-human IgE was used as the secondary antibody and NeutrAvidin HRP conjugate and substrate were used for signal production. M: molecular weight marker (kDa); CTL: control (unmodified egg white protein). Approximate locations for egg white allergens are indicated. Gray scale was used for gels and membranes and contrast was optimized to improve visualization.

The major EWPs ovotransferrin (76.6 kDa), ovalbumin (45 kDa) and lysozyme (14.3 kDa) ^8^ from both aggregate particles and unmodified EWP were separated by SDS-PAGE and identified by staining with CBB ( Figure 1A). An increase in molecular weight of ovotransferrin and ovalbumin, but not of lysozyme, was observed and this was polyphenol concentration dependent ( Figure 1A). In fact, NBT staining indicated that ovalbumin and ovotransferrin, but not lysozyme were modified by polyphenols and the degree of staining was dependent on the concentration of polyphenol ( Figure 1B). The staining also revealed several additional proteins poorly stained with CBB (indicated with stars), suggesting that the NBT staining of polyphenols more sensitively reveals the presence of protein than does CBB staining. As expected, control EWP did not react with NBT ( Figure 1B). The finding that polyphenols remain bound to proteins following SDS-PAGE and membrane transfer suggests a strong, perhaps covalent association between the molecules.

As shown in Figure 1C, ovotransferrin, ovalbumin and lysozyme in unmodified EWP were recognized by antigen-specific IgE antibodies from human plasma. However, for protein samples that contained polyphenols, ovotransferrin and ovalbumin as well as several of the proteins revealed by NBT but not CBB staining, appeared as white “ghost” bands ( Figure 1C). Generally, “ghost” bands occur when the substrate is depleted quickly by the enzyme at that location and ceases to produce light ^2^. Commonly, this is a result of a high concentration of one or more of the components of the enzymatic reaction. However, in this case, the phenomenon was not observed for the EWP control sample (which did not contain polyphenols) and increased with increasing amount of polyphenols, suggesting that the polyphenols are triggering the excessive consumption of substrate and appearance of the “ghost” bands. The phenomenon was also observed with other aggregate particles including whey protein isolate-green tea polyphenol and whey protein isolate-blueberry polyphenol aggregate particles (see Figure S1) indicating that “ghosting” was not dependent on specific EWPs. “Ghost” bands also occurred on a few blots in our previously published work, however, this did not affect data interpretation ^3, 9^. The same treatments were re-tested by fluorescence-based Western blotting and the data was consistent with that previously reported.

**Figure 2.**
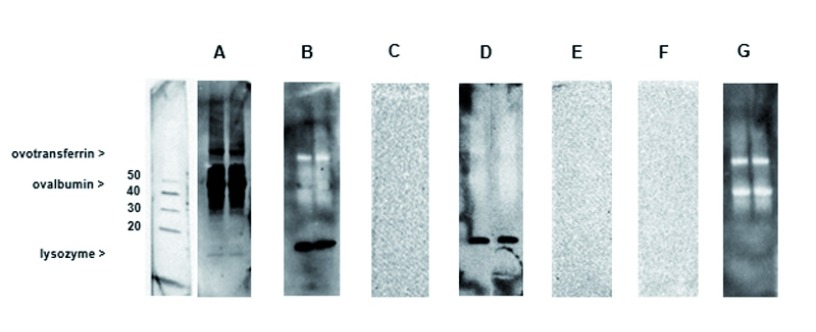
Evaluation of horseradish peroxidase hyperactivation by polyphenols. Western blot strips of ( **A**) unmodified egg white proteins and ( **B**– **G**) egg white protein-green tea polyphenol aggregate particles containing 15% total polyphenol content, after various immunoblotting treatments. ( **B**) received all immunoblotting reagents after membrane blocking - primary antibody (pooled human plasma from 7 egg white allergic individuals with egg white-specific IgE levels ranging from 15.4 to 100 kU L ^−1^), biotinylated goat IgG anti-human IgE secondary antibody, NeutrAvidin HRP conjugate, and substrate; ( **C**) the secondary antibody and NeutrAvidin HRP conjugate were omitted; ( **D**) the primary antibody was omitted and ( **E**) the primary and secondary antibody and NeutrAvidin HRP conjugate were omitted; ( **F**) the primary antibody and NeutrAvidin HRP conjugate were omitted and ( **G**) the primary antibody and secondary antibody were omitted. A molecular weight marker (kDa) is shown on the far left. Approximate locations for egg white allergens are indicated. Gray scale was used and contrast was optimized to improve visualization.

To further investigate the mechanism underlying “ghost” band formation on those blots, PVDF membrane-transferred unmodified and polyphenol-modified EWPs underwent treatment with a combination of different immunoblotting reagents. Results revealed that polyphenols promoted “ghost” band formation by interacting with HRP during HRP-substrate reactions ( Figure 2). “Ghost” bands were only observed on membrane strips containing green tea polyphenols and HRP ( Figure 2B, D, and G) and only HRP was required to produce “ghost” bands with polyphenol-modified EWPs ( Figure 2G). No “ghost” bands were observed when substrate alone was added to a membrane containing polyphenol-bound proteins ( Figure 2E). It should be noted that the light background in Figure 2C, E, and F is caused through a different mechanism than white “ghost” bands seen in B, D, and G. Since HRP is required for signal production, antibody-bound proteins on membranes not exposed to HRP ( Figure 2C, E, and F) were not detected, hence, the membrane appeared blank when imaged (grey spotting is an imaging artifact). In contrast, on membranes that were treated with HRP and contained polyphenols ( Figure 2B, D, and G), polyphenol-bound proteins appeared as white “ghost” bands due to depletion of locally available substrate and subsequent cessation of local light production. Interestingly, the lysozyme band was unaffected and apparently represents another artifact. This band did not require the presence of the primary antibody ( Figure 2D), indicating it occurs due to a non-specific reaction between the biotinylated goat IgG anti-human IgE secondary antibody-NeutrAvidin HRP conjugate and the substrate. Further, the intensity of this band increased in the presence of polyphenols ( Figure 2A, B and D), which seems contradictory since the NBT stain did not indicate polyphenols bound to lysozyme ( Figure 1B). It is possible that in the presence of polyphenols, specific binding of primary and therefore secondary antibodies to proteins may be reduced resulting in excess free secondary antibodies to bind lysozyme (which did not contain bound polyphenols).

Based on this experiment, exact mechanisms of HRP promotion by polyphenols cannot be determined. It is possible, based on the fact that polyphenols are able to act as “bridges” between proteins ^10^, that HRP non-specifically binds to protein-bound polyphenols at high concentrations, therefore rapidly depleting substrate (luminol) in close proximity to the enzyme. Further, it is possible that protein-bound polyphenols are able to promote HRP activity, as has been observed similarly with digestive enzymes ^11^. In both cases, this could result in the cessation of light emittance (depletion of locally available luminol).

It is important to note that the observations made in this study applied to a specific set of protein samples, secondary antibody, enzyme and chemiluminescence substrate. Other types of conjugated or unconjugated secondary antibodies, enzymes (e.g. alkaline phosphatase), or substrates have not been evaluated. However, while proper Western blot experimental designs include appropriate controls such as evaluation of unmodified proteins or antibody-antigen specificity, no control for protein-bound polyphenols as shown above has been described to date. The present study highlights the importance of evaluating polyphenol effects on chemiluminescence-based antigen detection in order to prevent false interpretation of data and reveals a new source of “ghost” band artifacts.

## Conclusion

We demonstrated that when attempting to evaluate IgE binding capacity of EWP-green tea polyphenol aggregate particles by enhanced chemiluminescence-based Western blotting, polyphenols which remained bound to egg white proteins after electrophoretic transfer to PVDF membrane created “ghost” bands in the presence of HRP. This study reveals protein-bound ligands as an unintended source of “ghost” band artifacts, and suggests that caution should be taken when evaluating polyphenol-bound proteins by enhanced chemiluminescence Western blotting.

Raw data for Figure 1. Protein distribution visualized by Coomassie Brilliant Blue staining (CBB), nitroblue tetrazolium (NBT) staining, and IgE binding capacity (Full legend and table are in the file). Click here for additional data file. Copyright: © 2017 Plundrich N et al. 2017 Data associated with the article are available under the terms of the Creative Commons Zero "No rights reserved" data waiver (CC0 1.0 Public domain dedication).

Raw data for Figure 2. Evaluation of horseradish peroxidase hyperactivation by polyphenols (Full legend and table are in the file). Click here for additional data file. Copyright: © 2017 Plundrich N et al. 2017 Data associated with the article are available under the terms of the Creative Commons Zero "No rights reserved" data waiver (CC0 1.0 Public domain dedication).

Raw data for Supplementary figure S1. Protein distribution, nitroblue tetrazolium (NBT) staining, and IgE binding capacity (Full legend and table are in the file). Click here for additional data file. Copyright: © 2017 Plundrich N et al. 2017 Data associated with the article are available under the terms of the Creative Commons Zero "No rights reserved" data waiver (CC0 1.0 Public domain dedication).

## Data availability

The data referenced by this article are under copyright with the following copyright statement: Copyright: © 2017 Plundrich N et al.

Data associated with the article are available under the terms of the Creative Commons Zero "No rights reserved" data waiver (CC0 1.0 Public domain dedication). 

**Dataset 1: Raw data for Figure 1. Protein distribution visualized by Coomassie Brilliant Blue staining (CBB), nitroblue tetrazolium (NBT) staining, and IgE binding capacity.** (Full legend and table are in the file).

DOI, 10.5256/f1000research.10622.d152366 ^12^

**Dataset 2: Raw data for Figure 2. Evaluation of horseradish peroxidase hyperactivation by polyphenols.** (Full legend and table are in the file).

DOI, 10.5256/f1000research.10622.d152367 ^13^

**Dataset 3: Raw data for Supplementary Figure S1. Protein distribution, nitroblue tetrazolium (NBT) staining, and IgE binding capacity.** (Full legend and table are in the file).

DOI, 10.5256/f1000research.10622.d162634 ^14^

## Supplementary material

**Figure S1: Protein distribution, nitroblue tetrazolium (NBT) staining, and IgE binding capacity.** (Full legend and table are in the file).

**Table S1: Replicate measurements of green tea extract for total phenolic content.** SD: standard deviation.
