# Supplementary material for: Protein-bound polyphenols create “ghost” band artifacts during chemiluminescence-based antigen detection
Source: F1000Res. 2017 May 26;6:254. Originally published 2017 Mar 13. [Version 2] doi: 10.12688/f1000research.10622.2 (PMC5497812; doi:10.12688/f1000research.10622.2)
Supplement: Raw data for Figure 1. Protein distribution visualized by Coomassie Brilliant Blue staining (CBB), nitroblue tetrazolium (NBT) staining, and IgE binding capacity — (Full legend and table are in the file). [file f1000research-6-12566-s0000.tgz › cde34e22-5c61-420b-8903-1c390d97caab_Raw_data_for_Figure_1.pdf]

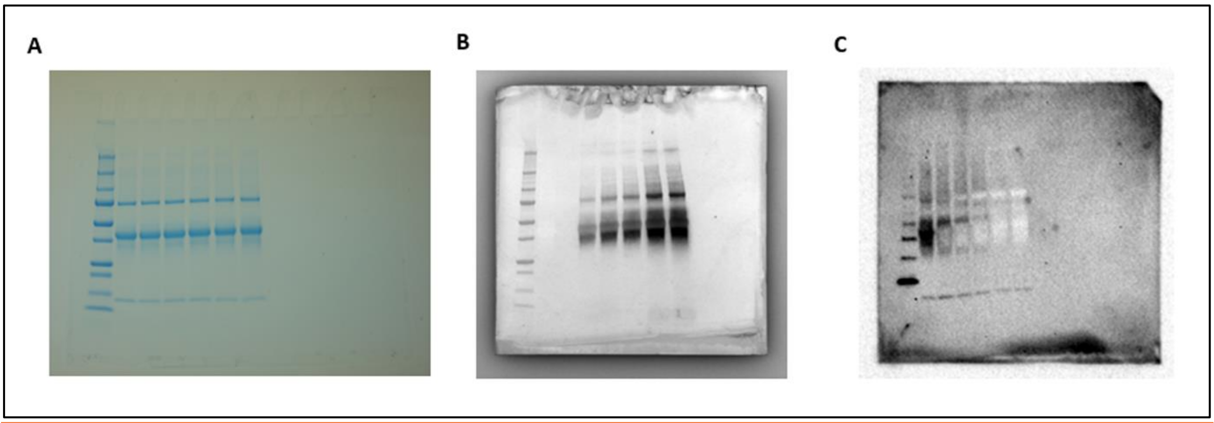

Raw data for Figure 1. Protein distribution visualized by Coomassie Brilliant Blue staining (CBB), nitroblue tetrazolium (NBT) staining, and IgE binding capacity. Contrast and color/greyscale was not adjusted and blot and gel images were not cropped. The image for Figure 1 A was taken with a camera (Cannon EOS III), the image for Figure 1 B was taken with a BioRad Gel Doc XR+ system, and the image for Figure 1 C was taken with a BioRad ChemiDoc MP system. (A) SDS-PAGE of unmodified egg white protein or egg white protein-polyphenol aggregate particles and stained with CBB; (B) Staining of green tea polyphenol-bound egg white proteins by NBT, following SDS-PAGE and subsequent electrophoretic transfer to a PVDF membrane; (C) corresponding Western blot. Pooled human plasma from 7 egg white-allergic individuals was used to bind antigens on the membrane. Egg white-specific IgE levels ranged from 15.4 to 100 kU L^{-1} as determined via ImmunoCAP (Phadia, Uppsula, Sweden). Biotinylated goat IgG anti-human IgE was used as the secondary antibody and NeutrAvidin HRP conjugate and substrate were used for signal production.
