# Supplementary material for: Protein-bound polyphenols create “ghost” band artifacts during chemiluminescence-based antigen detection
Source: F1000Res. 2017 May 26;6:254. Originally published 2017 Mar 13. [Version 2] doi: 10.12688/f1000research.10622.2 (PMC5497812; doi:10.12688/f1000research.10622.2)
Supplement: Raw data for Figure 2. Evaluation of horseradish peroxidase hyperactivation by polyphenols — (Full legend and table are in the file). [file f1000research-6-12566-s0001.tgz › 1a155b78-3bfe-43ab-869e-49325a270cf7_Raw_data_for_Figure_2.pdf]

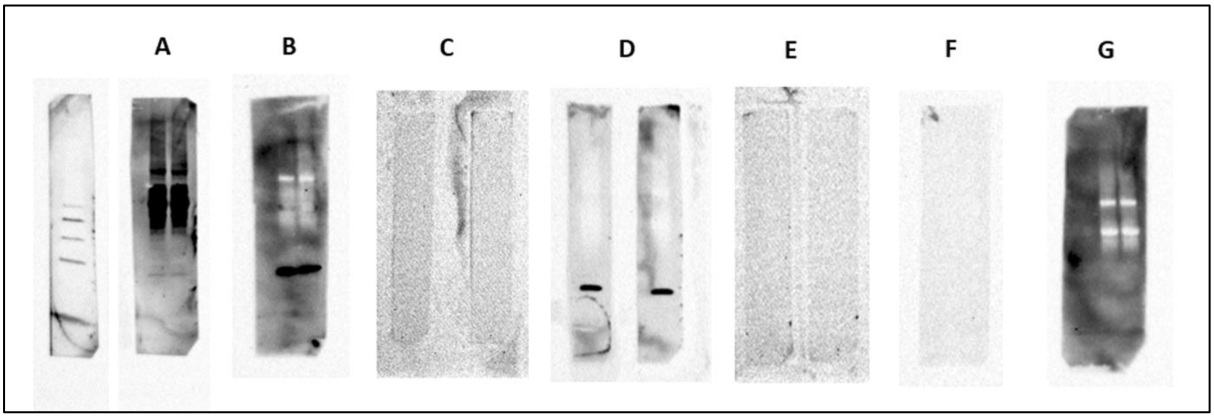

Raw data for Figure 2. Evaluation of horseradish peroxidase hyperactivation by polyphenols. Contrast and color/greyscale was not adjusted and blot and gel images were not cropped. Figure images were taken with a BioRad ChemiDoc MP system. Western blot strips of (A) unmodified egg white proteins and (B-G) egg white protein-green tea polyphenol aggregate particles containing 15% total polyphenol content, after various immunoblotting treatments. (B) received all immunoblotting reagents after membrane blocking - primary antibody (pooled human plasma from 7 egg white-allergic individuals with egg white-specific IgE levels ranging from 15.4 to 100 kU L^{-1}), biotinylated goat IgG anti-human IgE secondary antibody, NeutrAvidin HRP conjugate, and substrate; (C) the secondary antibody and NeutrAvidin HRP conjugate were omitted; (D) the primary antibody was omitted and (E) the primary and secondary antibody and NeutrAvidin HRP conjugate were omitted; (F) the primary antibody and NeutrAvidin HRP conjugate were omitted and (G) the primary antibody and secondary antibody were omitted.
