# Supplementary material for: Protein-bound polyphenols create “ghost” band artifacts during chemiluminescence-based antigen detection
Source: F1000Res. 2017 May 26;6:254. Originally published 2017 Mar 13. [Version 2] doi: 10.12688/f1000research.10622.2 (PMC5497812; doi:10.12688/f1000research.10622.2)
Supplement: Raw data for Supplementary figure S1. Protein distribution, nitroblue tetrazolium (NBT) staining, and IgE binding capacity — (Full legend and table are in the file). [file f1000research-6-12566-s0002.tgz › ae09eb0b-ae0a-47cc-a837-23187a0c7695_Raw_data_for_Supplementary_Figure_S1.pdf]

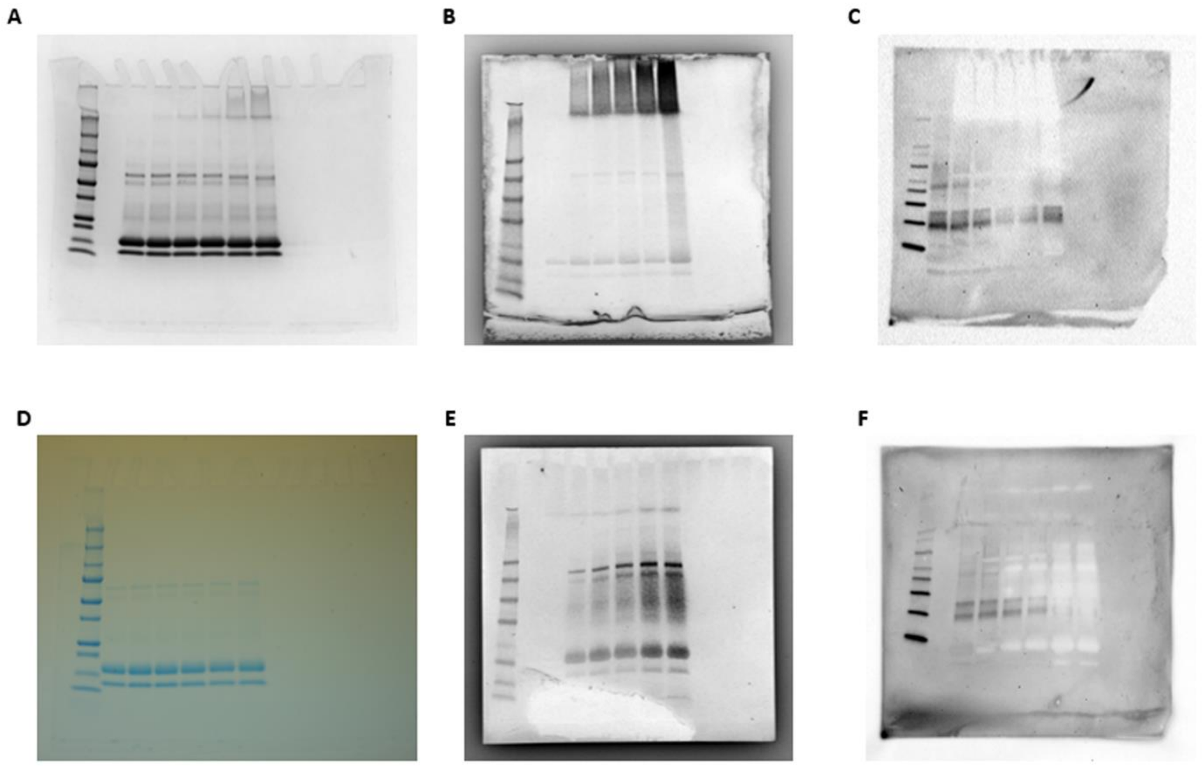

Raw data for Supplementary figure S1. Protein distribution, nitroblue tetrazolium (NBT) staining, and IgE binding capacity. Contrast and color/greyscale were not adjusted and blot and gel images were not cropped. Images for Figure S1 A, B and E were taken with a BioRad Gel Doc XR+ system, images for Figure S1 C and F were taken with a BioRad ChemiDoc MP system, and the image for Figure S1 D was taken with a camera (Cannon EOS III). (A) SDS-PAGE of unmodified whey protein isolate or whey protein isolate-blueberry polyphenol aggregate particles *(B)* Staining of blueberry polyphenol-bound whey proteins by NBT following SDS-PAGE and subsequent electrophoretic transfer to a PVDF membrane; (C) corresponding Western blot (pooled human plasma from 10 milk-allergic individuals was used to bind antigens on the membrane. Milk-specific IgE levels ranged from 17.1 to 100 kU L^{-1} as determined via ImmunoCAP (Phadia, Uppsula, Sweden). Biotinylated goat IgG anti-human IgE was used as the secondary antibody and NeutrAvidin HRP

conjugate and substrate were used for signal production.); *(D)* SDS-PAGE of unmodified whey protein isolate or whey protein isolate-green tea polyphenol aggregate particles. (E) Staining of green tea polyphenol-bound whey proteins by NBT following SDS-PAGE and subsequent electrophoretic transfer to a PVDF membrane; (F) corresponding Western blot. Pooled human plasma from 10 milk-allergic individuals was used to bind antigens on the membrane. Milk-specific IgE levels ranged from 17.1 to 100 kU L^{-1} as determined via ImmunoCAP (Phadia, Uppsula, Sweden). Biotinylated goat IgG anti-human IgE was used as the secondary antibody and NeutrAvidin HRP conjugate and substrate were used for signal production.
