# Supplementary material for: Protein-bound polyphenols create “ghost” band artifacts during chemiluminescence-based antigen detection
Source: F1000Res. 2017 May 26;6:254. Originally published 2017 Mar 13. [Version 2] doi: 10.12688/f1000research.10622.2 (PMC5497812; doi:10.12688/f1000research.10622.2)
Supplement: Supplementary file 4 [file f1000research-6-12566-s0003.tgz › 00a0844a-695b-403a-a1e2-316fda92e5e4.pdf]

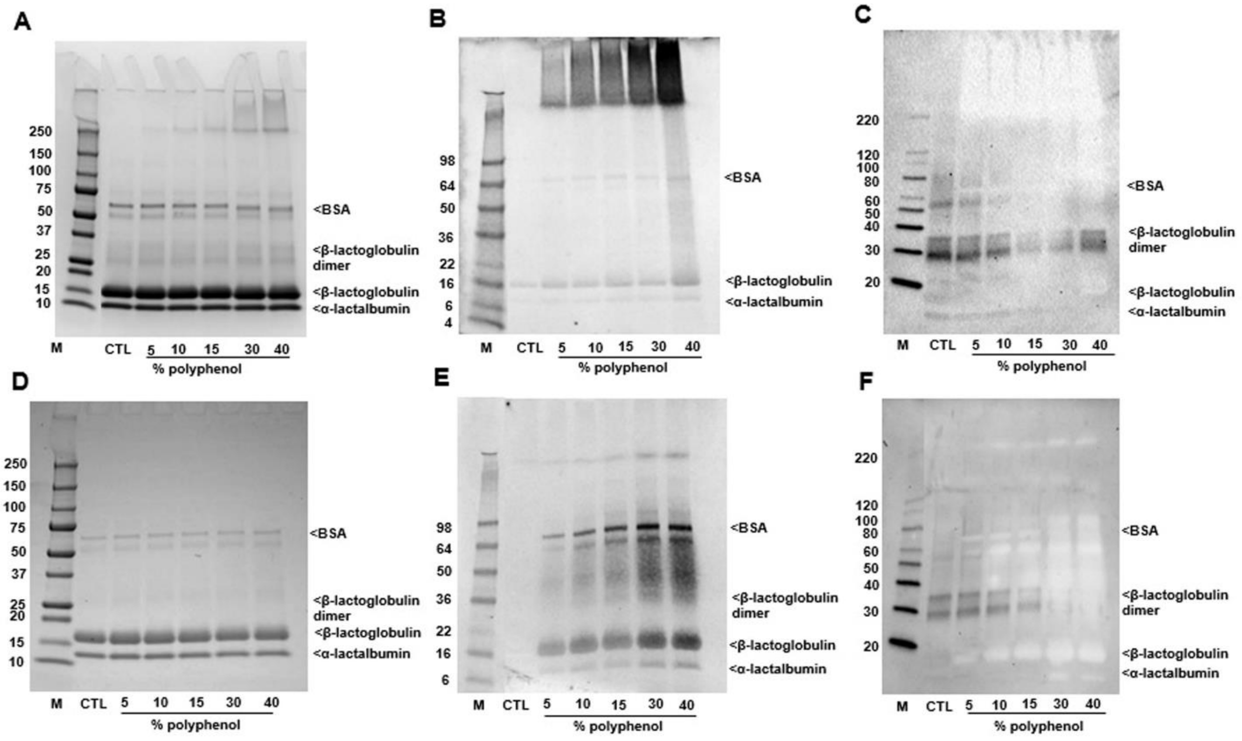

Figure S1. Protein distribution, nitroblue tetrazolium (NBT) staining, and IgE binding capacity. (A) SDS-PAGE of unmodified whey protein isolate or whey protein isolate-blueberry polyphenol aggregate particles containing 5, 10, 15, 30, and 40% polyphenols and stained with CBB. *(B)* Staining of blueberry polyphenol-bound whey proteins by NBT following SDS-PAGE and subsequent electrophoretic transfer to a PVDF membrane; (C) corresponding Western blot (pooled human plasma from 10 milk-allergic individuals was used to bind antigens on the membrane. Milk-specific IgE levels ranged from 17.1 to 100 kU L^{-1} as determined via ImmunoCAP (Phadia, Uppsula, Sweden). Biotinylated goat IgG anti-human IgE was used as the secondary antibody and NeutrAvidin HRP conjugate and substrate were used for signal production.); *(D)* SDS-PAGE of unmodified whey protein isolate or whey protein isolate-green tea polyphenol aggregate particles containing 5, 10, 15, 30, and 40% polyphenols and stained with CBB. (E) Staining of green tea polyphenol-bound whey proteins by NBT following SDS-PAGE and subsequent electrophoretic transfer to a PVDF membrane; (F) corresponding Western blot. Pooled human plasma from 10 milk-allergic individuals was used to bind antigens on the membrane. Milk-specific IgE

levels ranged from 17.1 to 100 kU L^{-1} as determined via ImmunoCAP (Phadia, Uppsula, Sweden). Biotinylated goat IgG anti-human IgE was used as the secondary antibody and NeutrAvidin HRP conjugate and substrate were used for signal production. M: molecular weight marker (kDa); CTL: control (unmodified whey proteins), BSA: bovine serum albumin. Approximate locations for whey allergens are indicated. Gray scale was used for gels and membranes and contrast was optimized to improve visualization. Lane 2 in panel A, E and F was empty and was removed from the image.

Beta-lactoglobulin dimer in unmodified whey protein isolate was recognized by antigen-specific IgE antibodies from human plasma (Fig 1C and F). However, BSA, β-lactoglobulin, and α-lactalbumin were only minimally or not at all detected. For protein samples that contained polyphenols, BSA, β-lactoglobulin, β-lactoglobulin dimer, and α-lactalbumin as well as several of the proteins revealed by NBT but not Coomassie Brilliant Blue staining, appeared as white “ghost” bands (Fig 1C and F). The phenomenon was not observed for the whey protein isolate control sample (which did not contain polyphenols) and increased with increasing amount of polyphenols. Beta-lactoglobulin and α-lactalbumin were recognized by NBT staining in the unmodified whey protein sample of panel B. Since there are no polyphenols present in this sample, this phenomenon could be due to artefactual staining of proteins containing free thiol groups as has previously been described [1]. However, only β-lactoglobulin contains one free thiol group, whereas α-lactalbumin does not contain any. The theory of thiol-group mediated artefactual staining cannot explain why α-lactalbumin was recognized by NBT.

References

1. Venugopal KSS and Adiga PR. Artifactual staining of proteins on polyacrylamide gels by nitroblue tetrazolium chloride and phenazine methosulfate. Anal Biochem. 1980; 101: 215-220.
