# Supplementary material for: Protein-bound polyphenols create “ghost” band artifacts during chemiluminescence-based antigen detection
Source: F1000Res. 2017 May 26;6:254. Originally published 2017 Mar 13. [Version 2] doi: 10.12688/f1000research.10622.2 (PMC5497812; doi:10.12688/f1000research.10622.2)
Supplement: Supplementary file 5 [file f1000research-6-12566-s0004.tgz › da881923-2f78-46d6-96db-2964888574ef.pdf]

Table S1. Replicate measurements of green tea extract for total phenolic content.

Sample	Total phenolic content in mg mL^{-1}	Mean total phenolic content in mg mL^{-1} ± SD
Green tea replicate measurement 1	36.74	36.76 ± 0.255
Green tea replicate measurement 2	37.02	
Green tea replicate measurement 3	36.51	

SD, standard deviation
