# Supplementary material for: Protein-bound polyphenols create “ghost” band artifacts during chemiluminescence-based antigen detection
Source: F1000Res. 2017 May 26;6:254. Originally published 2017 Mar 13. [Version 2] doi: 10.12688/f1000research.10622.2 (PMC5497812; doi:10.12688/f1000research.10622.2)
Supplement: Peer review history

# Peer review: Referee response for version 2

**DOI:** 10.5256/f1000research.12566.r23657

**Published:** 2017-07-03

**Authors:** Jerónimo Carnés

**Affiliations:** 1 Research & Development Department, Laboratorios LETI, Madrid, Spain

**General comments**

 The manuscript by Plundrich *et al.* describes that chemiluminiscence Western blotting with horseradish peroxidase modifies the signal when protein-bound polyphenols are investigated by this methodology. However the authors demonstrate that this signal is an artifact of the methodology , named "ghost band artifact". According to these results the authors confirm that the interpretation of the results must be investigated in detail. This is a relevant study because this methodology is commonly used, independently of the proteins and extracts investigated and it is useful for the understanding of the reaction and the interpretation of the results. The highlights of the article are clearly described in the manuscript.

 This reviewer considers that this findings are relevant when this methodology is going to be considered for the quantification of the proteins or when the objectives of these studies are to compare the concentration of different proteins in the same extract. In that case, the results obtained cannot be appropriate as a consequence of the different composition of the proteins (polyphenol aggregates particles). This topic should be discussed.

 Another point to take into account is the concentration of polyphenol aggregates in the proteins. According to the SDS-PAGE shown in the article (Figure 1A and 1B) and Western blot (Figure 1C), the highest concentrations are influencing significantly the image, however, the percentage of polyphenolic components that the authors are adding does not seem realistic in a common extract. This topic should be clarified and discussed.

 The authors should propose and include different alternatives with the objective to purify the natural extracts that contain these substances, by removing them, for example, using extensive dialysis or even treating the natural extracts . These studies could add value to the study.

**Minor comments**

 The introduction section should be focused and introduce the application.

 A reference should be add in paragraph 2. Previous studies have shown ghost bands… Please add what are these previous studies. Are they included in reference 3?

 Figure 2. Please add KDa to indicate that 20, 30, 40 etc correspond to this unit

 Methology should be detailed

 The authors should discus their experience with other extracts. Are they comparable? Probably it would be better to move their experience with peanut to the discussion section.

 The authors should propose different alternatives or at least include this topic in the discussion section.

I have read this submission. I believe that I have an appropriate level of expertise to confirm that it is of an acceptable scientific standard.

# Peer review: Referee response for version 2

**DOI:** 10.5256/f1000research.12566.r23049

**Published:** 2017-06-26

**Authors:** Christopher P Mattison

**Affiliations:** 1 Southern Regional Research Center, FPSQ (Food Processing and Sensory Quality Research Unit) , ARS (Agricultural Research Service), United States Department of Agriculture (USDA), New Orleans, LA, USA

.

I have read this submission. I believe that I have an appropriate level of expertise to confirm that it is of an acceptable scientific standard.

# Peer review: Referee response for version 1

**DOI:** 10.5256/f1000research.11446.r20891

**Published:** 2017-04-20

**Authors:** R Hal Scofield, Biji Kurien

**Affiliations:** 1 Arthritis and Clinical Immunology Program, Oklahoma Medical Research Foundation (OMRF) , Oklahoma City, OK, USA; 2 University of Oklahoma Health Sciences Center, Oklahoma City, OK, USA

This study assessed the binding capacity of IgE antibodies to egg white protein (EWP)-green tea polyphenol complex by enhanced chemiluminescence-based Western blotting method. The authors of this study found polyphenols that remained bound to egg white proteins following electrophoretic transfer to a PVDF membrane hyperactivated HRP, leading to the formation of “ghost” bands. Based on the results of this study the authors suggest caution when evaluating polyphenol-bound proteins by enhanced chemiluminescence Western blotting.

 While the article is of interest, this reviewer notes several concerns.

 The authors should take into consideration the possibility that polyphenols bound to the protein prevents binding of primary antibody and thus could produce these artifacts. Will the effect go away if using lower amounts of protein in each well (e.g. 1, 2 or 5 µg/well; the study currently uses 10 µg/well)? The authors have studied various combinations of immunoblotting reagents, including exclusion of primary antibody or use of only HRP-avidin (using EWP with 15% polyphenol) to study the reason for the formation of these “ghost” bands.  However, since there is the possibility of HRP-avidin interacting non-specifically with the antigen on the blot (in the absence of primary and secondary), the authors should try a system that does not involve the biotin-avidin system for increasing sensitivity of detection (just regular primary antibody, HRP secondary antibody and enhanced ECL detection). The authors should also try a non-chemiluminescence system to see if this problem could be reproduced (e.g. HRP with DAB detection).

 There are few other issues-

 Why is there a noticeable shift in lysozyme migration shown in Figure 1C if it does not bind polyphenols? Also, there is decreased detection of lysozyme in lanes with 30 and 40% polyphenols with the NBT system.

 Were the gels of different composition? The protein migration pattern appears different in Figures 1A, 1B and 1C. The use of a different molecular weight marker in Figure 1C probably accentuates this observed effect. Actually 5 different molecular weight markers have been used in this work (10 to 250 kD; 6 to 98 kD; 20-220 kD; 20 to 100 kD and 20 to 50 kD)!

 Figure S1A (and S1D), it is not clear how the proteins were stained? Were the proteins stained with Coomassie?

 In experiments shown in Figure 1C, the authors show that the “ghost” band increases with increasing amounts of polyphenol bound to the proteins. However, Figure S1C shows that there is no “ghost” band in the lane with β-lactoglobulin bound to 40% polyphenol, which is contrary to the hypothesis put forward by the authors.

 Do the authors have a reference to cite in support of the statement “Generally, “ghost” bands occur when the substrate is depleted quickly by the enzyme at that location and ceases to produce light”?

 The authors should consider re-writing the following sentences-

 “Following transfer of proteins by electroblotting from unmodified EWP and aggregate particles to a polyvinylidene difluoride (PVDF) membrane, the membrane was briefly hydrated in 100% methanol and polyphenol-modified proteins were detected with NBT and glycinate as described by Hagerman”

 “Transferred proteins from unmodified EWP served as a control and underwent full immunoblotting procedure”

We have read this submission. We believe that we have an appropriate level of expertise to confirm that it is of an acceptable scientific standard, however we have significant reservations, as outlined above.

# Response

**Published:** 2017-05-10

**Authors:** Mary Ann Lila

**Affiliations:** North Carolina State University, USA

*Comments to the Author:*

“This study assessed the binding capacity of IgE antibodies to egg white protein (EWP)-green tea polyphenol complex by enhanced chemiluminescence-based Western blotting method. The authors of this study found polyphenols that remained bound to egg white proteins following electrophoretic transfer to a PVDF membrane hyperactivated HRP, leading to the formation of “ghost” bands. Based on the results of this study the authors suggest caution when evaluating polyphenol-bound proteins by enhanced chemiluminescence Western blotting.

While the article is of interest, this reviewer notes several concerns.”

*The authors should take into consideration the possibility that polyphenols bound to the protein prevents binding of primary antibody and thus could produce these artifacts. Will the effect go away if using lower amounts of protein in each well (e.g. 1, 2 or 5 µg/well; the study currently uses 10 µg/well)?

Answer: Thank you. We have tested 5 µg/well for egg white protein-polyphenol complexes as well as for whey protein isolate-polyphenol complexes and observed “ghost” bands. We did not test even lower amounts of protein in this study. Our experiments have shown that “ghost” band formation appeared to be independent of primary antibody binding but dependent on the presence of HRP. However, it is possible that, in Figure 2B, the primary antibody was not able to bind but HRP, which was added as well, ultimately caused observed ”ghost” bands. Figure 1B did not allow us to determine if the primary antibody bound to proteins that appeared as “ghost” bands or not.

*The authors have studied various combinations of immunoblotting reagents, including exclusion of primary antibody or use of only HRP-avidin (using EWP with 15% polyphenol) to study the reason for the formation of these “ghost” bands.  However, since there is the possibility of HRP-avidin interacting non-specifically with the antigen on the blot (in the absence of primary and secondary), the authors should try a system that does not involve the biotin-avidin system for increasing sensitivity of detection (just regular primary antibody, HRP secondary antibody and enhanced ECL detection). The authors should also try a non-chemiluminescence system to see if this problem could be reproduced (e.g. HRP with DAB detection).

Answer: Thank you. We agree with the referee and in fact we now have moved on to fluorescence based detection in our recent studies. Experiments have shown no artifacts using this system. We wanted to move away from chemiluminescence based detection systems all together. 

There are few other issues-

*Why is there a noticeable shift in lysozyme migration shown in Figure 1C if it does not bind polyphenols? Also, there is decreased detection of lysozyme in lanes with 30 and 40% polyphenols with the NBT system.

Answer: Thank you for your comment. Figure 1B (NBT stain) shows that lysozyme was not detected at all. Or more specifically, the NBT stain revealed that lysozyme was not bound by polyphenols. The upward shift seen in Figure 1C is likely an artifact from either running the gel or occurred during the protein transfer onto the PVDF membrane (gel could have been shifted/skewed a bit during “sandwich” preparation in the iBlot electroblotting system). It can be seen that all lanes in Figure 1C appear to be skewed. Figure 1A (SDS-PAGE) shows an even run of lysozyme.

*Were the gels of different composition? The protein migration pattern appears different in Figures 1A, 1B and 1C. The use of a different molecular weight marker in Figure 1C probably accentuates this observed effect. Actually 5 different molecular weight markers have been used in this work (10 to 250 kD; 6 to 98 kD; 20-220 kD; 20 to 100 kD and 20 to 50 kD)!

Answer: Thanks. No, the same gels were used to create Figure 1 A, B and C (BioRad TGX mini protean precast gels 4-20%). Yes, the 10 to 250 kDa marker (BioRad Precision Plus) was used for gels, the 6 to 98 kDa marker (Invitrogen SeeBlue2) was used for NBT blots since this was the available marker at the time of data collection, and a 20-220 kDa marker (Invitrogen Magic Mark XP, an IgG labeled marker) was used for Western blots. We did not use a 20 to 100 kDa nor a 20 to 50 kDa marker. Only visible marker bands are shown alongside the Western blots, hence the possible confusion. 

*Figure S1A (and S1D), it is not clear how the proteins were stained? Were the proteins stained with Coomassie?

Answer: Thanks. Yes, they were also stained with Coomassie Brilliant Blue and we have now added this information to the respective figure legend.

*In experiments shown in Figure 1C, the authors show that the “ghost” band increases with increasing amounts of polyphenol bound to the proteins. However, Figure S1C shows that there is no “ghost” band in the lane with β-lactoglobulin bound to 40% polyphenol, which is contrary to the hypothesis put forward by the authors.

Answer: Thanks. The “ghost” band in Figure S1C is not very pronounced but can be seen for β-lactoglobulin.

*Do the authors have a reference to cite in support of the statement “Generally, “ghost” bands occur when the substrate is depleted quickly by the enzyme at that location and ceases to produce light”?

Answer: Thank you. Yes, we have now added a reference.

*The authors should consider re-writing the following sentences-

******

***“Following transfer of proteins by electroblotting from unmodified EWP and aggregate particles to a polyvinylidene difluoride (PVDF) membrane, the membrane was briefly hydrated in 100% methanol and polyphenol-modified proteins were detected with NBT and glycinate as described by Hagerman”***

Answer: Thanks. We have broken this rather long sentence into two, for greater clarity. It now reads: “Following transfer of proteins by electroblotting from unmodified EWP and aggregate particles to a polyvinylidene difluoride (PVDF) membrane, the membrane was briefly hydrated in 100% methanol. Subsequently, polyphenol-modified proteins were detected with NBT and glycinate as described by Hagerman”

***“Transferred proteins from unmodified EWP served as a control and underwent full immunoblotting procedure”***

Answer: Thanks. We have reworded for additional clarity. It now reads: “Transferred proteins from unmodified EWP served as controls. The proteins from unmodified EWP were subjected to the full immunoblotting procedure”.

# Peer review: Referee response for version 1

**DOI:** 10.5256/f1000research.11446.r20889

**Published:** 2017-03-23

**Authors:** Christopher P Mattison

**Affiliations:** 1 Southern Regional Research Center, FPSQ (Food Processing and Sensory Quality Research Unit) , ARS (Agricultural Research Service), United States Department of Agriculture (USDA), New Orleans, LA, USA

The findings of this manuscript should be published because the implications of the HRP findings on current and past research could be widespread.  The authors should be congratulated for taking the time and effort to examine the artifacts they observed rather than just ignoring them and moving on.  In its current form, however there are some important points that need to addressed in the manuscript to ease reader comprehension and focus the research on one topic or more clearly describe of the findings and implications of the 2 topics in the manuscript.   

1. What is the focus of the manuscript, the study of the effect of polyphenols on egg, or the artifact(s) resulting from the use of HRP?

- The title of the manuscript suggests the manuscript is focused to point out a potentially very serious and mis-leading artifact of using HRP for western blot signal generation, but the content of the text is mixed between pointing of the findings of the egg/tea polyphenol study findings and the HRP artifact
- I would argue that the HRP artifact is the primary purpose of the paper (as suggested in the title) and more in-depth discussion of the findings/implications is needed

1. Please consider re-writing the second paragraph of the introduction to sharpen the focus of the manuscript to coincide with the title…rather than the focus of green tea polyphenols on egg allergens.
2. In the introduction some discussion and referencing of ‘ghost bands’ from past publications would be useful and possibly a discussion of the topic of reciprocity failure (if relevant here) in signal generation?
3. Would the ghost bands be expected to obscure ‘real’ bands nearby or migrating at the same pace?
4. Did the authors notice these artifacts in their own past publications on similar topics?Plundrich *et al* 2014 ^1^. If so, this should be discussed and any discrepancies in their findings or conclusions that can be attributed to the HRP artifacts should be noted.
5. Findings using peanut allergens and tea (or other sources of) poly-phenols that lead to the same artifacts are important to point out.
6. Can the authors find a related published article/examples of other groups that may have suffered from the same artifact and mis-lead the authors of that research to put their findings in the context of other using the same reagents?
7. Could the authors please star/mark the bands that are considered “several additional proteins” that were detected with NBT staining but not CBB on Fig 1B?
8. Could the additional bands noted on Fig 1B represent oligomers/aggregates of the ovalbumin and ovotransferrin, and are these same bands present on the immunoblot?
9. Could you test directly the proposed interaction between HRP and green tea polyphenols observed by the blot in Fig 1C and Fig 2G?
10. Concerning the dark lysozyme band, this is a very important finding, but what evidence is there that this band is actually lysozyme? Are the authors aware of other examples of this non-specific artifacts with biotinylated 2ndary antibody-neutravidin-HRP complexes?
11. Consider changing the wording of the section; “due to a non-specific reaction between the secondary HRP-conjugated antibody” referring to the band in Fig 2D that I believe requires the secondary biotinylated antibody and the neutravidin-HRP conjugate.
12. In the conclusion the words “hyperactivated HRP” are mis-leading because there is no evidence of increased specific activity for the HRP so consider rewriting this sentence.

I have read this submission. I believe that I have an appropriate level of expertise to confirm that it is of an acceptable scientific standard, however I have significant reservations, as outlined above.

## References

[1] PlundrichNJKulisMWhiteBLGraceMHGuoRBurksAWDavisJPLilaMA: Novel strategy to create hypoallergenic peanut protein-polyphenol edible matrices for oral immunotherapy. *J Agric Food Chem*.2014;62(29) : 10.1021/jf405773b 7010-21 10.1021/jf405773b 24758688

# Response

**Published:** 2017-05-10

**Authors:** Mary Ann Lila

**Affiliations:** North Carolina State University, USA

Comments to the Author:

“The findings of this manuscript should be published because the implications of the HRP findings on current and past research could be widespread.  The authors should be congratulated for taking the time and effort to examine the artifacts they observed rather than just ignoring them and moving on.  In its current form, however there are some important points that need to addressed in the manuscript to ease reader comprehension and focus the research on one topic or more clearly describe of the findings and implications of the 2 topics in the manuscript. “  

*What is the focus of the manuscript, the study of the effect of polyphenols on egg, or the artifact(s) resulting from the use of HRP?

- ***The title of the manuscript suggests the manuscript is focused to point out a potentially very serious and mis-leading artifact of using HRP for western blot signal generation, but the content of the text is mixed between pointing of the findings of the egg/tea polyphenol study findings and the HRP artifact***
- ***I would argue that the HRP artifact is the primary purpose of the paper (as suggested in the title) and more in-depth discussion of the findings/implications is needed***

*Please consider re-writing the second paragraph of the introduction to sharpen the focus of the manuscript to coincide with the title…rather than the focus of green tea polyphenols on egg allergens.

Answer: Thank you. We re-wrote the second paragraph of the introduction in consideration of these points, to emphasize that the HRP artifact is the primary purpose of sharing these research results.

*In the introduction some discussion and referencing of ‘ghost bands’ from past publications would be useful and possibly a discussion of the topic of reciprocity failure (if relevant here) in signal generation?

Answer: Thanks. We have now included a sentence about previous studies.

*Would the ghost bands be expected to obscure ‘real’ bands nearby or migrating at the same pace?

Answer: Thank you. Based on our observations, no. However, major proteins we investigated were well separated. We may not be able to exclude the possibility of “real” bands to be obscured by a (especially strong) “ghost” band close by and/or migrating at the same pace.

*Did the authors notice these artifacts in their own past publications on similar topics? Plundrich et al 2014 ***^1^*** ***. If so, this should be discussed and any discrepancies in their findings or conclusions that can be attributed to the HRP artifacts should be noted.***

Answer: Thank. Yes, this was observed in Figure 2 of the Plundrich et al. 2014 paper (soluble fraction, top of blot shows high molecular weight material that appeared as a “ghost” band/smear). It was also observed in the Plundrich et al. 2015 paper, Figure 3 B (peanut protein-cranberry polyphenol complex) above Ara h 2 in the digestive samples (appears that smeary lanes appeared somewhat as “ghost” bands. In both cases, however, this did not affect findings made and conclusions drawn. In addition, the same treatments were re-tested using a new protocol (fluorescence Western blotting) and the data was consistent with that previously reported. We now included a sentence about this in the discussion.

*Findings using peanut allergens and tea (or other sources of) polyphenols that lead to the same artifacts are important to point out.

Answer: Thank you. Please see answer above.

*Can the authors find a related published article/examples of other groups that may have suffered from the same artifact and mislead the authors of that research to put their findings in the context of other using the same reagents?

Answer:  Thank you, this is a good question. At this time, we are not aware of any other studies that reported on similar artifacts such as those we found. After all, the detection method we used is one of many possible approaches.

*Could the authors please star/mark the bands that are considered “several additional proteins” that were detected with NBT staining but not CBB on Fig 1B?

Answer: Thanks. We have now indicated those additional proteins and the respective sentence in the text slightly rephrased.

*Could the additional bands noted on Fig 1B represent oligomers/aggregates of the ovalbumin and ovotransferrin, and are these same bands present on the immunoblot?

Answer: Thanks. The additional bands/smears observed are protein-polyphenol complexes/aggregates that have been revealed by the NBT stain. Coomassie Brilliant Blue also stains proteins that are complexed with polyphenols (see smears in Figure 1 A), however, the NBT stain more sensitively stains proteins that have been modified by polyphenols. Those protein complexes are also present on the immunoblot, however, most of them appeared as “ghost” bands.

*Could you test directly the proposed interaction between HRP and green tea polyphenols observed by the blot in Fig 1C and Fig 2G?

Answer: Thank you. This is a good question, and could be followed up on. We think green tea extract could directly be added to a PVDF membrane and similar experiments as in this study could be performed to test the direct effects between green tea polyphenols and HRP.

*Concerning the dark lysozyme band, this is a very important finding, but what evidence is there that this band is actually lysozyme? Are the authors aware of other examples of this non-specific artifacts with biotinylated 2ndary antibody-neutravidin-HRP complexes?

Answer: Thanks. The tentative identification of lysozyme was based on literature. Lysozyme was the only protein found in the 15 kDa range (MW~14 kDa; Desert et al. J Agric. Food Chem., 2001, 49: 4553–4561). We are not aware of other examples of this non-specific binding. However, it is possible that the biotin-moiety of the secondary antibody was able to bind to lysozyme, as has previously been observed by Green et al. (Nature, 1968, 217: 254-256), although this group described weak interactions. It is also possible that the secondary antibody concentration used was high and resulted in non-specific binding to lysozyme when other reagents were omitted.

*Consider changing the wording of the section; “due to a non-specific reaction between the secondary HRP-conjugated antibody” referring to the band in Fig 2D that I believe requires the secondary biotinylated antibody and the neutravidin-HRP conjugate.

Answer: Thanks. In fact, the “secondary HRP-conjugated antibody” refers to the “secondary biotinylated antibody that has been bound by neutravidin-HRP conjugate”. We have reworded the sentence to make it clear.

*In the conclusion the words “hyperactivated HRP” are misleading because there is no evidence of increased specific activity for the HRP so consider rewriting this sentence.

Answer: Thank you. We agree and have reworded this sentence.
